# 3D Organoid Culture From Adult Salivary Gland Tissues as an *ex vivo* Modeling of Salivary Gland Morphogenesis

**DOI:** 10.3389/fcell.2021.698292

**Published:** 2021-08-12

**Authors:** Donghyun Kim, Yeo-Jun Yoon, Dojin Choi, Jisun Kim, Jae-Yol Lim

**Affiliations:** Department of Otorhinolaryngology, Yonsei University College of Medicine, Seoul, South Korea

**Keywords:** salivary gland organoid, organoid culture, stem cells, retinoic acid, morphogenesis, lumen formation, vasoactive intestinal peptide

## Abstract

Lumen formation of salivary glands has been investigated using *in vivo* or *ex vivo* rudiment culture models. In this study, we used a three-dimensional (3D) salivary gland organoid culture system and demonstrated that lumen formation could be recapitulated in mouse SMG organoids. In our organoid culture system, lumen formation was induced by vasoactive intestinal peptide and accelerated by treatment with RA. Furthermore, lumen formation was observed in branching duct-like structure when cultured in combination of fibroblast growth factors (FGF) in the presence of retinoic acid (RA). We suggest RA signaling-mediated regulation of VIPR1 and KRT7 as the underlying mechanism for lumen formation, rather than apoptosis in the organoid culture system. Collectively, our results support a fundamental role for RA in lumen formation and demonstrate the feasibility of 3D organoid culture as a tool for studying salivary gland morphogenesis.

## Introduction

Murine salivary glands, particularly submandibular glands (SMGs), have been widely used as a model for studying salivary gland development and common processes in branching morphogenesis and tubulogenesis (Steinberg et al., 2005; Nitta et al., 2009; Hsu and Yamada, 2010; Knox et al., 2010; Nedvetsky et al., 2014). The process of duct formation encompasses duct elongation, fusion of microlumens to form contiguous large lumen, and lumen expansion during development stages (Nedvetsky et al., 2014). Several factors, including the fibroblast growth factor (FGF) family, epidermal growth factor (EGF) family, Wnt pathway components, bone morphogenetic proteins (BMPs), neurotrophic growth factor family, and neurotransmitter signaling components, are involved in the tightly regulated salivary gland development in a spatiotemporal manner (De Moerlooze et al., 2000; Miyazaki et al., 2004; Jaskoll et al., 2005; Knox et al., 2010; Patel et al., 2011; Mattingly et al., 2015).

Retinoids are a group of nutritional compounds that include retinol, retinal, and retinoic acid (RA). RA has several roles in organ morphogenesis, embryonic development, immune system, and growth of various cell types (Tanumihardjo, 2011). RA-mediated signaling activates its nuclear receptors, including the retinoic acid receptor (RAR) family and retinoid X receptor (RXR) family transcription factors. RAR or RXR isoforms have redundant roles, enabling them to compensate for each other under specific conditions. RA has been reported to be an essential factor in the initial stages of embryonic development of SMGs in the salivary glands (Wright et al., 2015; Abashev et al., 2017; Metzler et al., 2018). However, due to the limitations of using experimental models, the mechanism of adult mouse SMG tissue morphogenesis, including lumen formation, has remained elusive.

Recent advances in 3D organoid culture have enabled the establishment of long-term stem cell-based organotypic cultures (Clevers, 2016; Kretzschmar and Clevers, 2016), and research has demonstrated that organoid cultures can recapitulate *in vivo* morphogenesis of tissues, making them of use as model systems for investigating organ development and regeneration. Salivary gland organoids can be derived from murine embryonic stem cells (Tanaka et al., 2018), murine adult stem cells (Maimets et al., 2016), or human adult stem cells (Pringle et al., 2016). However, the application of salivary gland organoids in studies of adult tissue morphogenesis remains unexplored. Therefore, in this study, we postulated that a salivary gland organoid culture system could recapitulate lumen formation during SMG morphogenesis, and investigated whether SMG organoids could be harnessed to elucidate the mechanism of lumen formation.

## Materials and Methods

### Mice

Six to sixteen-week-old female C3H mice were purchased from Jackson Laboratory (Bar Harbor, ME) and maintained under specific pathogen-free conditions in a facility accredited by AAALAC International. All experiments were approved by the Institutional Animal Care and Use Committee of Yonsei University College of Medicine (Approval Number; 2017-0092).

### Cell Isolation and Organoid Culture

Mouse salivary gland organoids were generated from the SMGs of 6–16-week-old female C3H mice. SMG tissues were minced with a razor blade, and the homogenate was incubated in digestion buffer consisting of Hanks’ balanced salt solution (HBSS, Biowest, Nuaille, France), 0.63 mg/mL of collagenase type II (Worthinton Biochem, Lakewood, NJ, United States), 0.5 mg/mL of hyaluronidase (Sigma-Aldrich, St. Louis, MO, United States), and 6.25 mM CaCl_2_ (Sigma-Aldrich) for 1 h at 37°C in a shaking incubator. After incubation, debris was filtered through a series of 100-, 70-, and 40-μm strainers (SPL Life Sciences Co., Ltd., Seoul, South Korea). The isolated cells were mixed with 20 μL of growth factor reduced (GFR) Matrigel (Corning, NY, United States) and seeded in 48-well plates (Greiner, Kremsmünster, Austria). The basal medium contained advanced DMEM/F12 (Thermo Fisher Scientific, Waltham, MA, United States) with penicillin/streptomycin (Gibco, Grand Island, NY, United States), HEPES (Biowest), and GlutaMAX (Gibco). The basal medium was supplemented with Primocin (0.1 mg/mL, InvivoGen, San Diego, CA, United States), N-acetyl cysteine (1.25 mM, Sigma-Aldrich), B27 minus vitamin A (Gibco), EGF (5 nM, Gibco), bFGF (1 nM, PeproTech, Rocky hill, NJ, United States), 10% R-spondin 1-conditioned media (homemade), 50% Wnt3A-CM (homemade) or 5% afamin/Wnt3A-CM (MBL-life science, Woburn, MA, United States), and Y-27632 (10μM, Tocris, Bristol, United Kingdom) to prepare the SMG organoid growth medium. The medium (250 μL/well) was added and changed every 2–3 days. The organoids were maintained at 37°C in a humidified atmosphere under 5% CO_2_.

### Immunofluorescence Microscopy

For formalin-fixed paraffin-embedded immunofluorescence analysis, sections were deparaffinized and rehydrated. The sections were subjected to heat antigen retrieval (10 mM Tris, 1 mM EDTA, 0.05% Tween-20) for 40 min. After cooling the slide in ice water, the sections were blocked for 1 h with 5% normal goat serum (NGS, Jackson ImmunoResearch Laboratories, West Grove, PA, United States) or 5% normal donkey serum (NDS, Jackson ImmunoResearch Laboratories), depending on the secondary antibody. For whole-mount organoid immunofluorescence analysis, the organoids were fixed with either 4% paraformaldehyde for 30–60 min or ice cold acetone/methanol (1:1) for 1 min followed by 0.3% Triton X-100 in phosphate-buffered saline (PBS). The organoids were blocked with 5% NGS or NDS in 0.05% PBS-Tween 20 for 2 h at 4°C. The sections or organoids were incubated with primary antibodies overnight at 4°C as follows: chicken anti-KRT5 (905901, BioLegend, 1:1,000); mouse anti-KRT7 (ab9021, Abcam, 1:200); rabbit anti-KRT-19 (ab52625, Abcam, 1:500); rabbit anti-CD133 (orb99113, Biorbyt, 1:50); rabbit anti-VIPR1 (AVR-001, Alomone, 1:100); mouse anti-MIST1 (ab110919, Abcam, 1:50), goat anti-ACTA2 (NB300-978, Novus Bio, 1:1,000), rabbit anti-cleaved Caspase-3 (9661s, CST, 1:500), and goat anti-TJP1 (ABIN6254231, antibodies-online. 1:1,000) in tris-buffered saline (TBS) or phosphate-buffered saline PBS. On the following day, the sections or organoids were rinsed thrice in TBS or PBS for 5 min (sections) or 30 min (whole-mount). The primary antibodies were detected using 488-, 555-, 647-conjugated secondary antibodies (Invitrogen), and the nuclei were counter stained using Hoescht 33342 (1:1,000, Invitrogen). Fluorescence was analyzed using the Carl Zeiss LSM 700 confocal microscope and Zen software.

### Bright-Field Image Acquisition and Analysis

Bright-field images (40× or 100× magnification) of each culture were acquired using the Nikon Eclipse Ti 2-U microscope with NIS-Elements BR software (Nikon, Tokyo, Japan). The number of total organoids and organoids with lumen were counted manually.

### Quantitative Reverse Transcription-PCR (qRT-PCR)

Total RNA was isolated from the cultured organoids using TRIzol Reagent (Invitrogen) and processed for reverse transcription using the PrimeScript RT Reagent Kit (Takara) according to the manufacturer’s protocol. Gene expression was assessed by the conventional SYBR method using the QuantStudio 5 Real-Time PCR Systems (Applied Biosystems). Primer information for analysis of mouse genes was as follows: *Vipr1*: GCA GCA AGA TGT GGG ACA ACC T (Forward), CAG TTG TGA CCA GCC TTC TTC AG (Reverse); *Vipr2*: TGC CTC TTC AGG AAG CTG CAC T (Forward), TGG AGT AGA GCA CGC TGT CCT T (Reverse); *Vip:* GAT GCC GTT TGA AGG AGC AGG T (Forward), GAA GTC TGC TGT AAT CGC TGG TG (Reverse); *Rarb*: GCT TCG TTT GCC AGG ACA AGT C (Forward), TGG CAT CGG TTC CTA GTG ACC T (Reverse); *Krt5*: TTG GTG TTG GCA GTG GCT TT (Forward), CCC GCT ACC CAA ACC AAG AC (Reverse); *Krt7*: GCT CTC GCT CCA CTG CTT AC (Forward), CGC CAG CAA GCT CTG ATT GA (Reverse); *Aqp5*: GCC ACA TCA ATC CGG CCA TT (Forward), GGG CTG CCA CGT AGA AGA TG (Reverse); *Acta2*: GCC ATC ATG CGT CTG GAC TT (Forward), ATC TCA CGC TCG GCA GTA GT (Reverse); *Gapdh*: AGG GCA TCT TGG GCT ACA CT (Forward), CGG CAT CGA AGG TGG AAG AG (Reverse). Gene expressions were normalized to the expression of *Gapdh*.

### Flow Cytometry

To assess cell death in mouse SMG organoids, we used the EzWay Annexin V-FITC Apoptosis Detection Kit (KOMA Biotech, Seoul, South Korea). Briefly, mouse SMG organoids were dissociated with TrypLE Express (Thermo Fisher Scientific) for 20 min at 37°C, and incubated in 100μL of 1 × annexin V binding buffer with annexin V-FITC and PI for 15 min at titrated concentrations. After incubation, 400μL of binding buffer was added, and the samples were subsequently subjected to flow cytometry using LSRFortessa X-20 (BD Biosciences). Data were collected and analyzed using FlowJo software (Tree Star Inc., Ashland, OR, United States).

### Statistical Analysis

All experiments were performed at least three times. Data are presented as the mean with standard mean error (SEM). Unpaired two-tailed Student’s *t*-tests (two groups) and one-way ANOVA (more than two groups) with Tukey’s *post hoc* test were performed using GraphPad Prism 7 (GraphPad Software Inc., CA). Values of ^∗^*p* < 0.05, ^∗∗^*p* < 0.01, and ^∗∗∗^*p* < 0.001 were considered statistically significant, while *p* > 0.05 was not considered statistically significant.

## Results

### Salivary Gland Organoids Can Recapitulate Lumen Formation During Salivary Gland Morphogenesis

**FIGURE 1:**
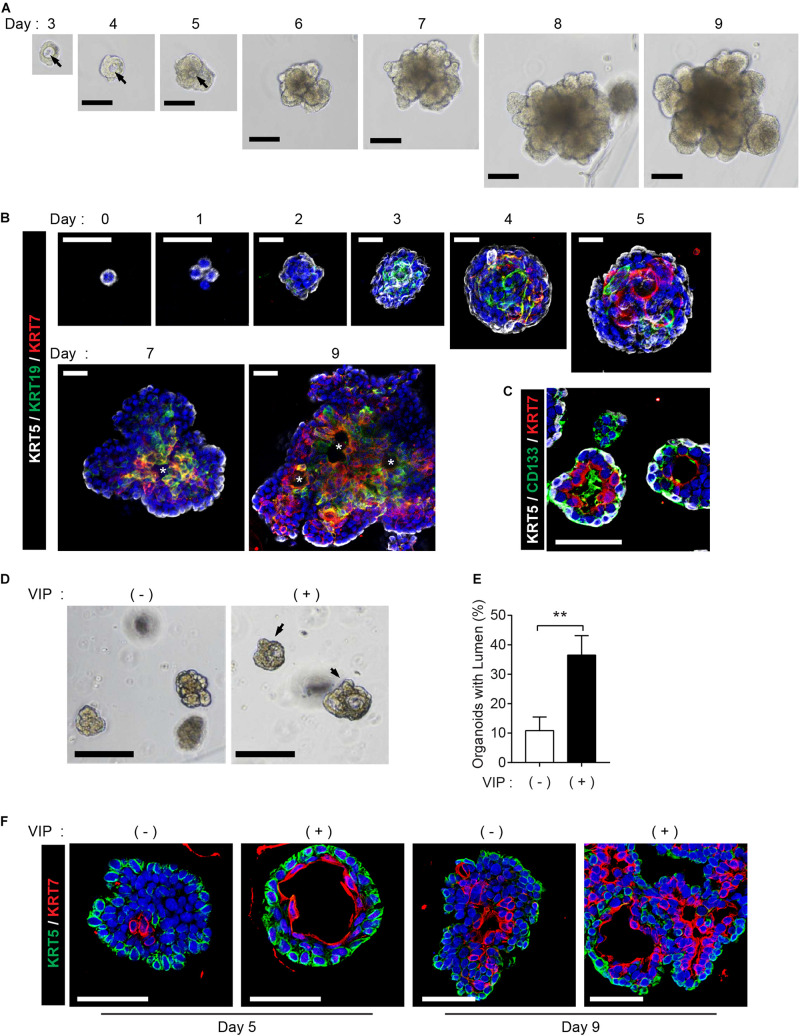
Recapitulation of lumen formation during SMG development in a mouse SMG organoid culture. **(A)** Organoid growth from single cells was monitored at each time point. Black arrows indicate internal lumen formation as shown in the bright-field microscopy images. Scale bar indicates 100 μm. **(B)** SMG organoids were harvested at each time point and subjected to whole-mount immunofluorescence microscopy for the evaluation of KRT5 (white), KRT19 (green), and KRT7 (red) expressions from days 0 to 9. Up to day 5, images were obtained through maximum intensity projection. From days 7 to 9, single images were obtained from z-stack. Micro-lumens were denoted with white asterisks. Scale bars indicate 20 μm. Nuclei were counterstained with DAPI (blue). **(C)** KRT5 (white), CD133 (green), and KRT7 (red) expressions on day 4. Scale bars indicate 50 μm. Nuclei were counterstained with DAPI (blue). **(D)** The organoids were treated with 200 nM of VIP on day 2, and lumen enlargement was observed on day 4. Black arrows indicate internal lumen formation. Scale bars indicate 100 μm. **(E)** The proportion of organoids with lumen were determined (*n* = 3). The results are expressed as the mean ± SD. **p < 0.01. **(F)** SMG organoids were harvested on days 5 and 9, and 200 nM of VIP was treated on the last 1 day. Organoid were subjected to immunofluorescence staining for KRT5 (green) and KRT7 (red). Scale bars indicate 50 μm. Nuclei were counterstained with DAPI (blue). All experiments were performed three times independently.

The mouse SMG organoids were generated and maintained under specific conditions according to a previously reported protocol (Maimets et al., 2016). We could detect microlumen formation from day 3 of the organoid culture, while lumen in organoids was not clearly seen in brightfield when cultured for longer periods (Figure 1A). Next, we investigated whether the cell constitution and organoid characteristics recapitulate the process of lumen formation during morphogenesis. First, we monitored the temporal expression kinetics of KRT5, KRT19, KRT7, and CD133, which are known as markers of basal cells, luminal cells, terminally differentiated luminal cells, and apical membranes of luminal cells, respectively, in developing mouse SMGs (Walker et al., 2008; Nedvetsky et al., 2014). On day 0, the organoids consisted of KRT5^+^KRT19^–^KRT7^–^ basal ductal cells (Figure 1B). The organoid culture led to the expression of KRT19^+^ cells on day 3, and also started to express KRT7 on day 4. In addition, from day 5, cells expressing KRT19 and cells expressing KRT7 began to be distinguished from organoids, and microlumen which is not contiguous was clearly found on days 7 and 9 (Figure 1B, Supplementary Figure 1A, and Supplementary Video 1). After 4 days of culture, the organoids contained CD133^+^ luminal cells (Figure 1C), which is similar to a presumptive lumen reminiscent of mouse SMG development (Nedvetsky et al., 2014). These data indicated that luminal cells in this condition were terminally differentiated. However, other factors were still required for contiguous large lumen formation. Lumen formation and expansion are known to occur in the presence of vasoactive intestinal protein (VIP) in mouse SMGs (Nedvetsky et al., 2014). Therefore, we determined whether lumen formation could be achieved with the addition of VIP into the organoid culture medium. After 2 days of VIP treatment, the microlumens were enlarged and clearly visible, compared to those in the non-treated organoids (Figure 1D). Although the addition of VIP did not affect the number of organoids (data not shown), we confirmed that VIP promoted the formation of lumens (Figure 1E). Interestingly, VIP not only accelerated lumen formation at day 5 but also induced the formation of contiguous lumen with a defined expression of basal/luminal ductal cell markers on day 9 (Figure 1F). Basal cells and luminal cells were clearly distinguished in the VIP-treated organoids compared to the non-treated organoids. The expression of the basal marker gene, Krt5, was not altered; but on day 9, luminal marker genes, *Krt19* and *Krt7*, were somewhat decreased after VIP treatment, suggesting that VIP-induced lumen formation occurred after transcriptional regulation (Supplementary Figure 1B). Collectively, these results implied that SMG organoid culture can recapitulate lumen formation during SMG morphogenesis in the presence of a niche factor, VIP. In addition, VIP induces clear differentiation between luminal and basal cells in mSMG organoids.

### RA Signaling Enhances VIP-Mediated Lumen Formation in Salivary Gland Organoids

**FIGURE 2:**
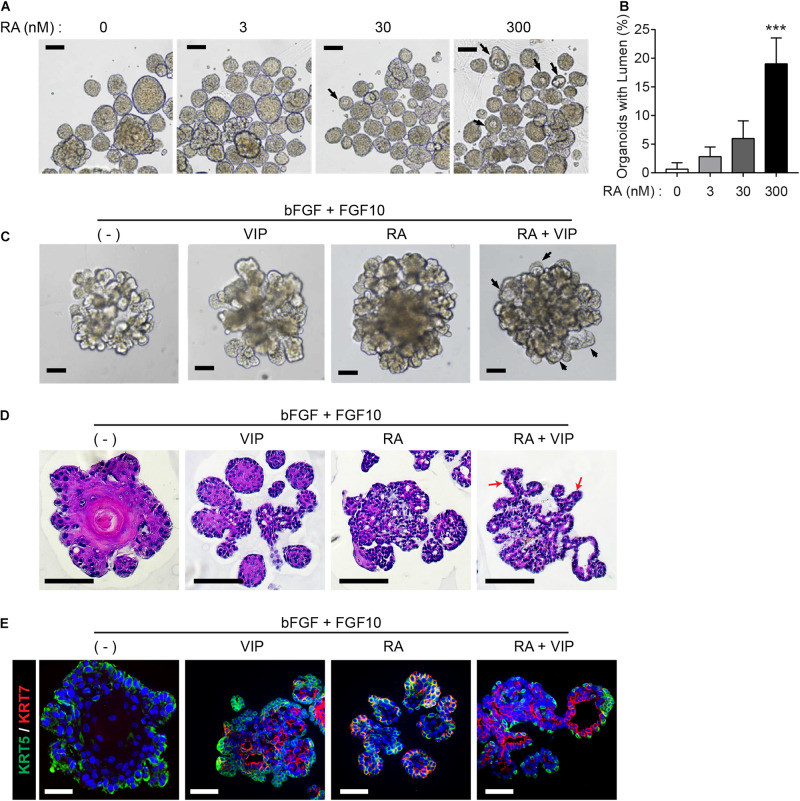
Retinoic acid promotes lumen formation in SMG organoids. **(A)** SMG organoids were cultured with different doses of RA and treated with 200 nM VIP on day 4. Microscopic images of lumen formation were obtained on day 6. Scale bars indicate 100 μm. **(B)** The proportion of organoids containing internal lumen were calculated, followed by statistical analysis (*n* = 3). Results are expressed as the mean ± SD. ***p < 0.001. **(C–E)** SMG organoids were cultured with or without RA in combination with 1 nM bFGF and 5 nM FGF10 throughout the culturing period. At day 9, organoids were either unstimulated or stimulated with VIP for 1 day. **(C)** Branching ducts were observed with brightfield microscopy. Black arrows indicate formation of lumens near end buds. Scale bars indicate 100 μm. **(D)** Harvested organoid were subjected to H&E staining to observe lumen formation in branching ducts (red arrows). Scale bars indicate 100 μm. **(E)** Organoids were subjected to immunofluorescence staining for KRT5 (green) and KRT7 (red). Scale bars indicate 50 μm. Nuclei were counterstained with DAPI (blue). All experiments were performed three times independently.

Next, we explored whether RA plays a pivotal role in lumen formation in our SMG organoid culture system. Since retinoids are present in serum trace quantities in Wnt3A-conditioned medium (Wnt3A-CM) (Kim et al., 1996), further experiments were conducted with serum-free afamin/Wnt3A to exclude the possibility of retinoids in Wnt3A-CM affecting lumen formation (Mihara et al., 2016). In order to examine the effect of RA and VIP on lumen formation of SMG organoids, the SMG organoids were cultured with different doses of RA and treated with 200 nM VIP on day 4. RA alone did not induce distinct differences in lumen formation in the absence of VIP in the culture medium (data not shown), whereas the addition of VIP to the organoid culture on day 4 in the RA-containing culture medium increased the proportion of the lumen-containing organoids (Figures 2A,B). Next, we maintained the organoid culture with combination of FGF ligands, which are known to be involved in budding and branching morphogenesis in salivary gland development (Hoffman et al., 2002; Steinberg et al., 2005), in order to induce lumen formation in organoids with more complex structure containing several branches and buds. Prior to culture using the combination of FGF, we tested whether the difference in morphology caused by FGFs in the mouse SMG development stage could be reproduced in organoids (Supplementary Figure 2A). In the absence of FGFs, small and round-shaped organoids were formed. In the presence of bFGF, short ducts with endbuds were observed, while long ducts were observed in the presence of FGF10. When bFGF and FGF10 were co-treated, complex types of organoids were observed (Supplementary Figure 2A). It was also found that basal and luminal cells were present in all conditions (Supplementary Figure 2B). Therefore, we decided to treat bFGF and FGF10 and tested whether RA or VIP also affected lumen formation in organoids with complex morphology. As expected, brightfield images showed that both RA and VIP treatment induced lumen expansion in branching organoids, while this phenotype was severely compromised upon the removal of RA (Figure 2C). Furthermore, lumen formation in branching ducts was observed in H&E-stained images when treated with both RA and VIP (Figure 2D). However, when treated with VIP only, branching ducts were scarcely detected, suggesting that RA may promote branching of adult SMG (Wang et al., 2006). In addition, RA and VIP promoted the compartmentalization of luminal cells and basal cells, as assessed by immunofluorescence staining for KRT5 and KRT7 (Figure 2E). Collectively, these results suggested that RA is necessary for terminal luminal cell differentiation in salivary gland organoids.

### RA Signaling Induces Lumen Formation via VIPR Signaling

**FIGURE 3:**
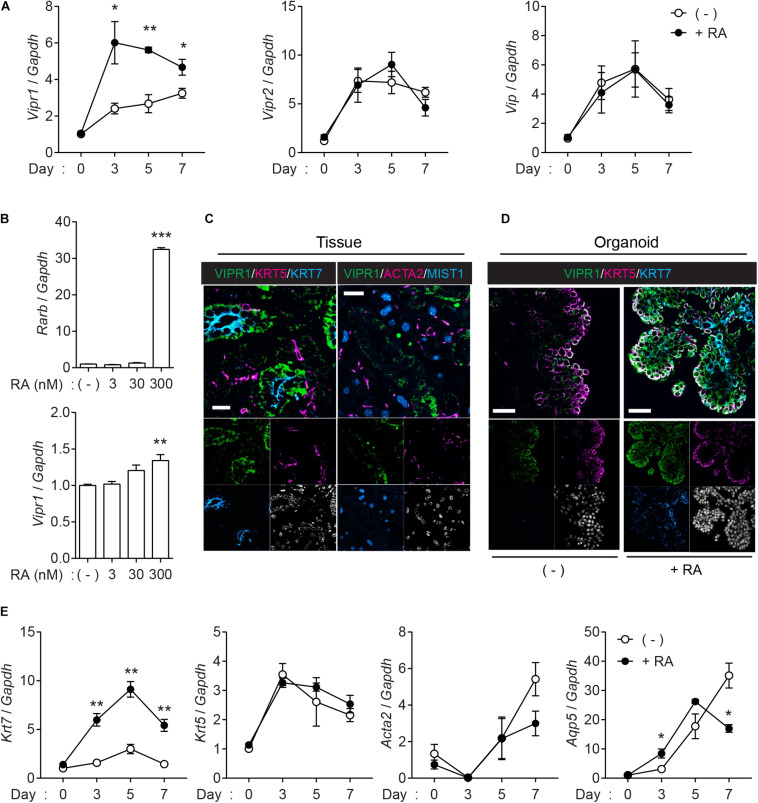
Retinoic acid induces *Vipr1* gene expression. **(A)** SMG organoids were treated with or without RA (300 nM) for the indicated time. After harvesting, the organoids were subjected to qRT-PCR to evaluate gene expression associated with VIP signaling (*n* = 4). **(B)** SMG organoids were treated with varying doses of RA for 7 days and subjected to qRT-PCR for the evaluation of gene expression of *Vipr1* and *Rarb*, a positive control, for RA-mediated signaling (*n* = 4). **(C)** Expression of VIPR1 (green) in mouse SMG tissues (left) was assessed by co-staining with KRT5 (magenta, left), KRT7 (cyan, left), ACTA2 (magenta, right), and MIST1 (cyan, right). Nuclei were counterstained with DAPI (white) and each single channel image was placed below. Scale bars indicate 20 μm. **(D)** The mouse SMG organoids cultured with (bottom) or without RA (top) were subjected to immunofluorescence for the evaluation of VIPR1 (green), KRT5 (magenta), and KRT7 (cyan) expressions. Nuclei were counterstained with DAPI (white), and each single channel image was placed below. Scale bars indicate 50 μm. **(E)** The expression of several salivary gland markers in organoids treated with or without RA were analyzed using qRT-PCR at the indicated time points (*n* = 4). Results are expressed as the mean ± SEM. *p < 0.05, **p < 0.01, and ***p < 0.001. All experiments were performed at least three times independently.

Next, we assessed whether VIPR signaling is upregulated in the organoids when treated with RA. Quantitative real-time polymerase chain reaction (qRT-PCR) confirmed that *Vipr1* expression increased in the presence of RA (Figure 3A), whereas *Vipr2*, another receptor for VIP, and *Vip* ligand elicited no significant difference compared to the expression in non-treated groups during organoid culture. Induced *Vipr1* expression began from day 3 and continued until day 7, which corresponds with the period of internal lumen formation detected in immunofluorescence staining, as shown in Figure 1B. To test the dose-dependent induction of *Vipr1*, mouse SMG organoids were treated with different doses (0–300 nM) of RA. Only 300 nM of RA significantly induced *Rarb*, a positive control for RA signaling, and *Vipr1* expression (Figure 3B), indicating that sufficient amounts of RA are required for the *Vipr1* expression.

Immunofluorescence staining was performed to confirm whether the upregulated mRNA of *Vipr1* was translated to the expression of VIPR1. Immunofluorescence staining of mouse SMG tissues showed that VIPR1 was present in the basolateral membrane of KRT7^+^ luminal cells; however, it was not present in MIST1^+^ acinar or ACAT2^+^ myoepithelial cells in murine SMG tissues (Figure 3C). In SMG organoids, KRT7^+^ cells on the luminal side co-expressed VIPR1 in the presence of RA, although VIPR1 was also expressed in KRT5^+^ basal cells of organoid culture (Figure 3D). MIST1^+^ acinar cells were not present in the organoids, and ACTA2^+^ myoepithelial cells were present, but VIPR1 was not co-expressed (Supplementary Figure 3). Next, we sought to determine whether RA treatment affects the expression of other salivary gland markers in SMG organoids. Consistent with protein expressions, RA treatment markedly increased the expression of *Krt7*; however, it did not alter the expression of *Krt5* or *Acta2*. The expression of *Aqp5* (acinar marker) decreased in late time points, compared to non-treated groups (Figure 3E), suggesting that RA might regulate acinar cell differentiation. Collectively, these results suggested that RA signaling induces lumen formation in mouse SMG organoids via the induction of VIPR1 expression and KRT7^+^ luminal cell differentiation.

### RAR-Mediated Regulation of *Vipr1* Promotes Lumen Formation in Mouse SMG Organoids

**FIGURE 4:**
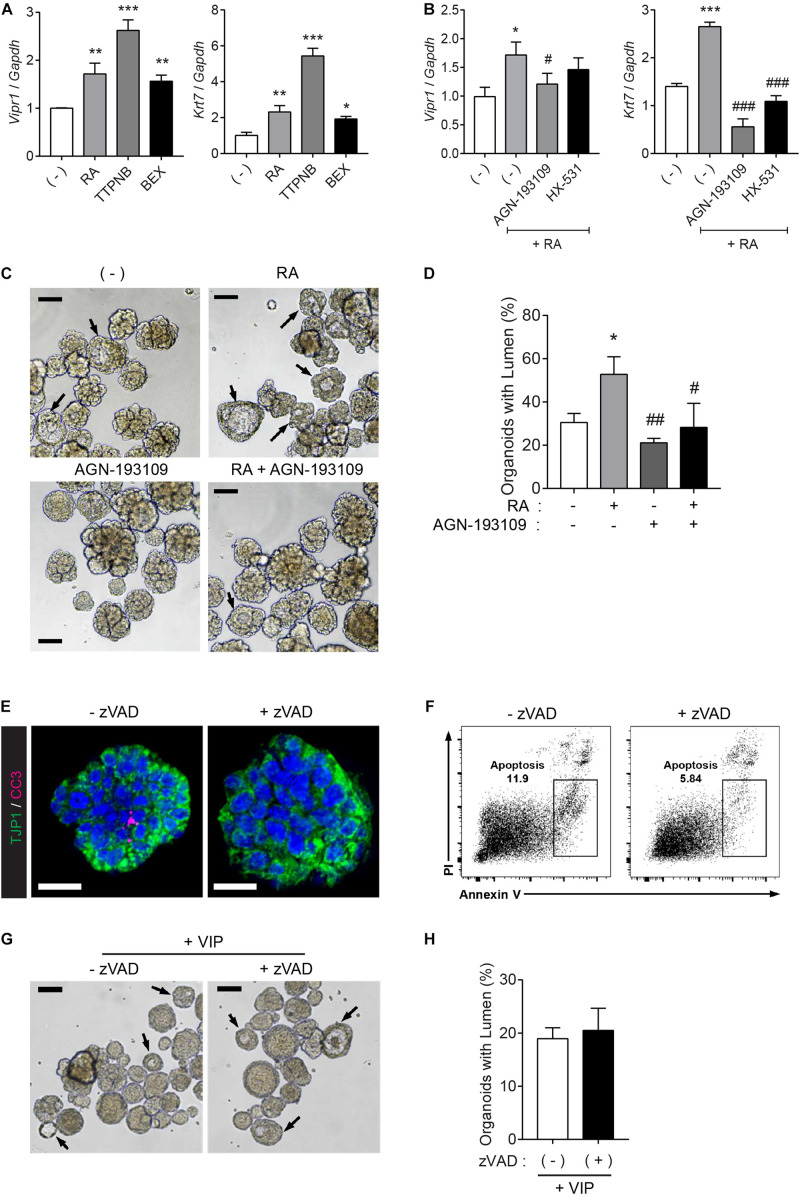
RAR activation induces lumen formation in SMG organoids. **(A)** SMG organoids were cultured with 300 nM RA, TTNPB (RAR agonist), or bexarotene (RXR agonist) for 7 days and subjected to qRT-PCR for the detection of *Vipr1* and *Krt7* expression (*n* = 4). **(B)** SMG organoids were treated with RA (300 nM), RA + 3 μM AGN-193109 (RAR antagonist), or RA + 3 μM HX (HX-531, RXR antagonist) throughout the culturing period and subjected to qRT-PCR for the detection of *Vipr1* and *Krt7* expression (*n* = 4). **(C–D)** SMG organoids were treated with RA (300 nM), AGN-193109 (3 μM), or both. The organoids were treated with VIP (200 nM) on day 2 for lumen formation. After 3 days, lumen formation was observed via microscopic images **(C)**. Black arrows indicate organoids containing visible lumen. The proportion of organoids with lumen were calculated and statistically analyzed **(D)** (*n* = 3). Scale bars indicate 100 μm in **(C)**. **(E)** SMG organoids were treated with 50μM z-VAD-FMK, a pan-caspase inhibitor, for 2 days, followed by whole-mount immunostaining with antibodies against CC3 (cleaved caspase-3) and TJP1 (ZO-1). DAPI (blue) was used for counterstaining the nucleus. Scale bars indicate 20 μm. **(F)** Apoptotic cell death (Annexin-V^+^ PI^–^) in SMG organoids treatedwith or without zVAD was examined using flow cytometry. **(G)** Representative bright-field images of organoids with lumen were obtained on day 5. Black arrows indicate organoids containing visible lumen. Scale bars indicate 100 μm. **(H)** The proportion of organoids with lumen were calculated (*n* = 3). Results are expressed as the mean ± SEM. *p < 0.05, **p < 0.01, and ***p < 0.001 when compared to the non-treated groups. *^#^p* < 0.05, *^##^p* < 0.01, and *^###^p* < 0.001 when compared to the RA-treated groups. All experiments were performed three times independently.

To investigate which receptor is pivotal to *Vipr1* expression and lumen formation, the organoids were treated with RAR agonist (TTNPB) or RXR-selective agonist (bexarotene) instead of RA, since RA binds with either nuclear RAR or RXR transcription factors and controls downstream gene expression by binding to DNA sequences of response elements (Dawson and Xia, 2012; Cunningham and Duester, 2015). The RA-mediated *Vipr1* and *Krt7* expressions were higher when treated with RAR-selective agonist at the same concentration, indicating that these genes are regulated in an RAR-dependent manner (Figure 4A). To confirm RAR-dependent gene regulation, RAR or RXR-selective antagonists (AGN-193109 and HX-531, respectively) were treated along with RA in SMG organoids. We found that the RAR antagonist significantly reduced RA-mediated gene expression, although partial RXR-mediated gene regulation was also observed (Figure 4B). Next, we determined whether RAR-dependent gene induction could affect lumen formation. Consistent with our hypothesis, lumen formation in SMG organoids was promoted by RA treatment and was severely compromised when treated with RAR antagonist (Figures 4C,D). Collectively, we suggest that RA promotes lumen formation through the induction of luminal cell-related gene expression in a RAR-dependent manner.

### RA Signaling Induces Lumen Formation in an Apoptosis-Independent Manner

It has been suggested that the underlying mechanisms of lumen formation in mouse SMGs involve the apoptosis of central epithelial cells in presumptive ducts and endbuds by depleting FGF signals (Jaskoll and Melnick, 1999; Melnick and Jaskoll, 2000; Teshima et al., 2016). RA has been reported to antagonize FGFs during development (Cunningham et al., 2013). Therefore, we examined whether lumen formation by RA can be attributed to apoptosis of the inner cells. To do so, the organoid cultures were treated with a pan-caspase inhibitor, z-VAD-fmk (zVAD), to inhibit apoptosis. Immunofluorescence results showed that zVAD inhibited naturally occurring apoptosis in the organoids (Figure 4E). Moreover, zVAD-mediated inhibition of apoptosis was confirmed using flow cytometry (Figure 4F). However, bright-field images of the SMG organoids showed that pre-treatment of zVAD had no effect on VIP-induced lumen formation (Figure 4G and its quantitative analysis in Figure 4H). Increased zVAD concentration did not inhibit VIP-induced lumen formation (data not shown). These results indicated that RA induces lumen formation in an apoptosis-independent manner.

## Discussion

The salivary glands play important roles in providing lubrication for chewing and swallowing, digestion, and vocalization, as well as protection against microbial infections (Tucker, 2007; Mattingly et al., 2015). Salivary gland hypofunction caused by radiation therapy, aging, or autoimmune diseases, severely impairs an individual’s oral health and quality of life (Jensen et al., 2010; Rocchi and Emmerson, 2020). Stem cell-based regeneration therapy has been regarded as a potential strategy to overcome salivary gland hypofunction, and translational research investigating salivary gland regeneration based on adult stem cell therapies is being intensively performed. The importance of understanding salivary gland morphogenesis has been emphasized along with the importance of gaining mechanistic insights into the regeneration of salivary glands using adult stem cells (Lombaert and Hoffman, 2010; Patel and Hoffman, 2014). In this study, we demonstrated that RA induces lumen formation in adult mouse SMGs and this adult organoid culture system is capable of recapitulating salivary gland morphogenesis. These findings have important implications on our understanding of salivary gland morphogenesis in adult tissues, and provide an opportunity to harness this organoid culture system as a tool for studying salivary gland regeneration or modeling.

Previous studies on salivary glands using adult stem cells have exploited floating culture or nanoscaffold microwells to enhance progenitor cell proliferation and stem cell properties (Shin et al., 2018; Koslow et al., 2019). However, these salisphere or spheroid cultures are more comparable to cell aggregations, which make it difficult to recapitulate complex tissue morphogenesis and the surrounding extracellular matrix (ECM). To overcome these limitations, an organoid system cultured in serum-free and biochemically defined media with ECM (e.g., laminin-111 or Matrigel) has been suggested as an alternative to 3D culture. This 3D organoid culture system facilitates the investigation of not only personalized medicine and disease models but also the developmental processes of organs using adult stem cells (Fatehullah et al., 2016; Xu et al., 2018). To extend the potential of salivary gland organoids as a tool for investigation of tissue morphogenesis, it is important to study the essential niche factors required to develop a 3D organoid culture system for efficient tissue recapitulation.

VIP, the ligand for VIPR1, was thought to regulate not only luminal cell proliferation but also lumen formation, which is mediated by the cAMP/PKA pathways, respectively (Knox et al., 2010; Nedvetsky et al., 2014). Although RA alone showed limited effects on lumen formation, we demonstrated that RA promotes lumen formation in the presence of VIP, suggesting that lumen formation is induced by VIP and accelerated by RA treatment. Furthermore, we discovered that RA promotes lumen formation in mouse SMG organoids by the induction of VIPR1 and KRT7 expression, and that RA-induced lumen formation is achieved via VIPR1-RAR mediated signaling, not via apoptosis pathways, as zVAD treatment did not affect lumen formation in SMG organoids. We suggest that the RA-mediated induction of *Krt7* expression can be explained by the direct effects of RA or indirect outcomes of lumen formation, indicating that further investigation is needed to completely understand the function of RA in salivary gland development based on our organoid system.

In mouse SMG tissues, RA is synthesized and supplied from the mesenchyme surrounding the developing salivary gland epithelium. RA signaling begins in the early developmental stages and is activated throughout the morphogenetic process of the glands (Wright et al., 2015; Metzler et al., 2018). Intriguingly, we found that RA promoted lumen formation in our organoid culture, but also inhibited differentiation into acinar cells. Although the regulation of acinar cell differentiation by RA in other exocrine glands, including lacrimal gland and pancreas, has been studied (Ubels et al., 2002; Tulachan et al., 2003), the detailed regulatory mechanisms of RA in the differentiation of acinar cells in the development of salivary glands remain unknown. Therefore, further studies will be required to elucidate the negative regulation of RA in the differentiation of salivary gland acinar cells. As niche factors can be added or withdrawn from an organoid culture, our culture system provides a model for the investigation of the temporal requirements of niche factors in tissue development; for example, separate utilization of the growth and differentiation media would enable efficient tissue recapitulation (Huch et al., 2015). Although our system is based on adult stem cells, due to some similarities between embryonic development and adult tissue regeneration (Nedvetsky et al., 2014; Emmerson et al., 2017), there is a possibility that our findings are applicable to embryonic stages. Moreover, our findings can be extended to other culture systems, including embryonic stem cells, iPSC, or tissue explant culture, to explore the role of RA in the development or regeneration of salivary glands.

In conclusion, we found that the administration of RA to organoid culture medium leads to lumen formation via the induction of *Vipr1* and *Krt7* expressions, and that RA promotes VIP-mediated lumen expansion in a RAR-dependent manner. Our study suggests that salivary gland organoid culture is a useful system for investigating the niche factors associated with salivary gland morphogenesis.

## Data Availability Statement

The original contributions presented in the study are included in the article/Supplementary Material, further inquiries can be directed to the corresponding author/s.

## Ethics Statement

The animal study was reviewed and approved by the Institutional Animal Care and Use Committee of Yonsei University College of Medicine (Approval Number; 2017-0092).

## Author Contributions

DK and Y-JY designed the study, performed the experiments, analyzed the data, and wrote the manuscript. DC and JK performed H&E staining and immunofluorescence staining. J-YL supervised the project and wrote the manuscript. All authors revised the article and approved the submission of revised article.

## Conflict of Interest

The authors declare that the research was conducted in the absence of any commercial or financial relationships that could be construed as a potential conflict of interest.

## Publisher’s Note

All claims expressed in this article are solely those of the authors and do not necessarily represent those of their affiliated organizations, or those of the publisher, the editors and the reviewers. Any product that may be evaluated in this article, or claim that may be made by its manufacturer, is not guaranteed or endorsed by the publisher.

## Supplementary Material

Supplementary Figure 1 Expressions of basal and luminal cell markers in salivary gland organoids. **(A)** Maximum intensity projected images of organoid at day 7 (up) and day 9 (down). Organoids were subjected to immunofluorescence for KRT5 (white), KRT19 (green), KRT7 (red), and DAPI (blue). Scale bar indicates 50 μm. **(B)** SMG organoids were harvested on days 5 and 9, and 200 nM of VIP was treated on the last 1 day. Harvested organoids were subjected to qRT-PCR to evaluate the expressions of *Krt5*, *Krt19*, and *Krt7* (*n* = 3). Results are expressed as the mean ± SEM. ^∗^*p* < 0.05.

Supplementary Figure 2 FGF-induced complex structure is observed in salivary gland organoids. SMG organoids were cultured in combination with 1 nM bFGF and 5 nM FGF10 throughout the culture. **(A)** FGF-induced changes in morphology were observed via brightfield microscopy in salivary gland organoids. Scale bar indicates 500 μm. **(B)** Organoids were subjected to immunofluorescence for KRT5 (green) and KRT7 (red). Nuclei were counterstained with DAPI (blue). Scale bar indicates 50 μm.

Supplementary Figure 3 VIPR1 expression in salivary gland organoid. The mouse SMG organoids cultured with RA were subjected to immunofluorescence for the evaluation of VIPR1 (green), ACTA2 (magenta), and MIST1 (cyan) expressions. Nuclei were counterstained with DAPI (white), and each single channel image was placed below. Scale bars indicate 50 μm.
