# Supplementary material for: 3D Organoid Culture From Adult Salivary Gland Tissues as an ex vivo Modeling of Salivary Gland Morphogenesis
Source: Front Cell Dev Biol. 2021 Aug 12;9:698292. doi: 10.3389/fcell.2021.698292 (PMC8397473; doi:10.3389/fcell.2021.698292)
Supplement: Supplementary Figure 1 — Expressions of basal and luminal cell markers in salivary gland organoids. (A) Maximum intensity projected images of organoid at day 7 (up) and day 9 (down). Organoids were subjected to immunofluorescence for KRT5 (white), KRT19 (green), KRT7 (red), and DAPI (blue). Scale bar indicates 50 μm. (B) SMG organoids were harvested on days 5 and 9, and 200 nM of VIP was treated on the last 1 day. Harvested organoids were subjected to qRT-PCR to evaluate the expressions of Krt5, Krt19, and Krt7 (n = 3). Results are expressed as the mean ± SEM. ∗p < 0.05. [file Data_Sheet_1.pdf]

Supplementary Figures

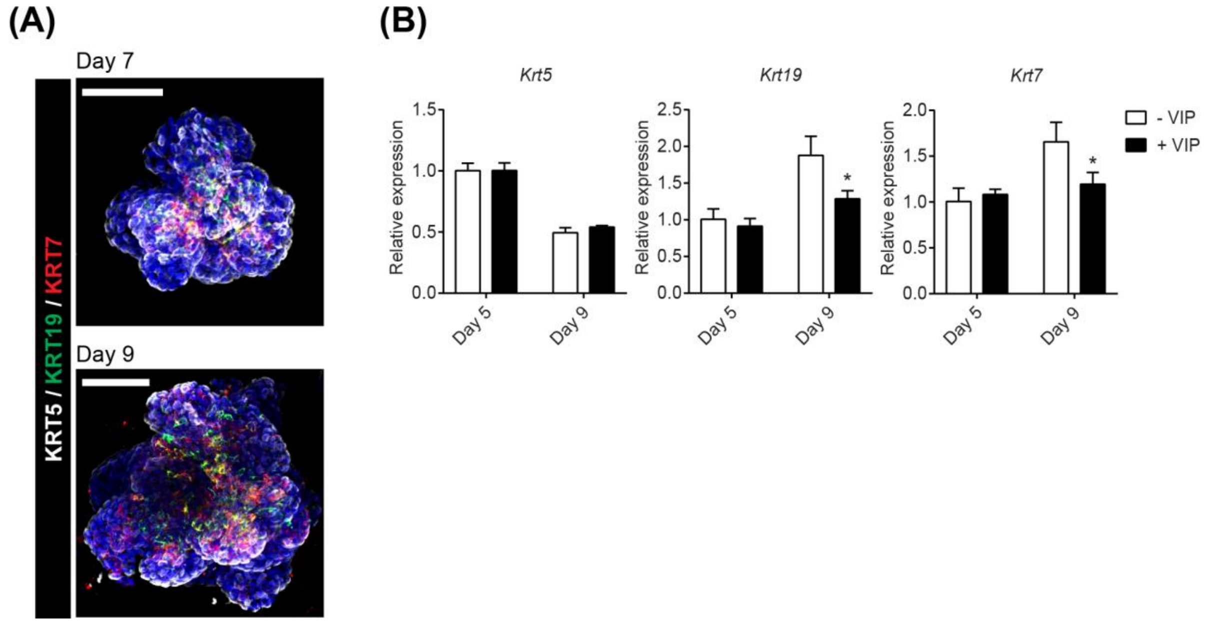

Figure S1. Expressions of basal and luminal cell markers in salivary gland organoids.

(A) Maximum intensity projected images of organoid at day 7 (up) and day 9 (down). Organoids were subjected to immunofluorescence for KRT5 (white), KRT19 (green), KRT7 (red), and DAPI (blue). Scale bar indicates 50 μm.

(B) SMG organoids were harvested on day 5 and day 9. 200 nM of VIP was treated on the last 1 day. Harvested organoids were subjected to qRT-PCR to evaluate the expressions of *Krt5*, *Krt19*, and *Krt7* ($n = 3$). Results are expressed as the mean ± S.E.M. * $p < 0.05$

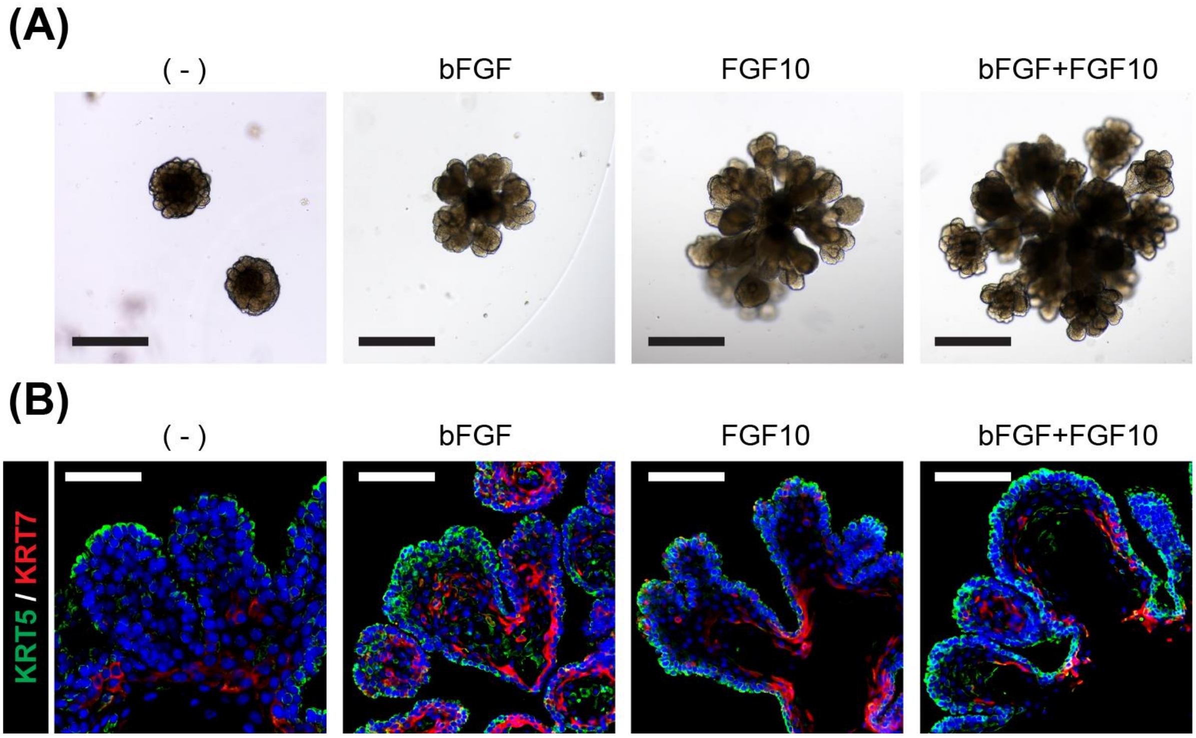

Figure S2. FGF-induced complex structure is observed in salivary gland organoids.

SMG organoids were cultured in combination with 1 nM bFGF and 5 nM FGF10 throughout the culture.

(A) FGF-induced changes in morphology were observed via brightfield microscopy in salivary gland organoids. Scale bar indicates 500 μm.

(B) Organoids were subjected to immunofluorescence for KRT5 (green) and KRT7 (red). Nuclei were counterstained with DAPI (blue). Scale bar indicates 50 μm.

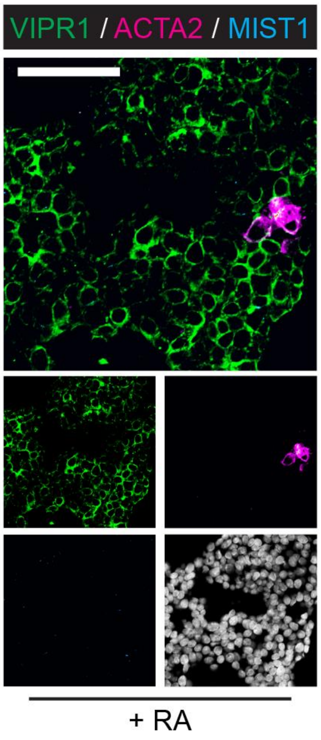

Figure S3. VIPR1 expression in salivary gland organoid.

The mouse SMG organoids cultured with RA were subjected to immunofluorescence for the evaluation of VIPR1 (green), ACTA2 (magenta), and MIST1 (cyan) expressions. Nuclei were counterstained with DAPI (white), and each single channel image was placed below. Scale bars indicate 50 μm.

Video S1. Time-lapse video from z-stack images of organoid at day 9

Organoids were subjected to immunofluorescence for KRT5 (white), KRT19 (green), KRT7 (red), and DAPI (blue). Scale bar indicates 20 μm.
